# *Granulicatella adiacens* infections in children: a single-center retrospective study

**DOI:** 10.1007/s10096-025-05154-0

**Published:** 2025-05-08

**Authors:** Türkan Aydın Teke, Ayşe Kaman, Fatma Nur Öz, Zeynep Gökçe Gayretli Aydın, Hülya Şeker Yıkmaz, Gülsüm İclal Bayhan

**Affiliations:** 1 Department of Pediatric Infectious Disease, Ankara Etlik City Hospital, Ankara, Türkiye; 2 Department of Pediatric Infectious Disease, Faculty of Medicine, Karadeniz Technical University, Trabzon, Türkiye; 3 Department of Pediatrics, Faculty of Medicine, Ankara Yıldırım Beyazıt University, Ankara City Hospital, Ankara, Türkiye; 4 Department of Pediatric Infectious Disease, Faculty of Medicine, Ankara Yıldırım Beyazıt University, Ankara City Hospital, Ankara, Türkiye

**Keywords:** *Granulicatella adiacens*, Bacteraemia, Endocarditis, Central line-related bloodstream infection, Children

## Abstract

**Purpose:**

*Granulicatella* spp. although rare, are notable pathogens in various infections, particularly in bacteraemia and infective endocarditis. Because of their unique growth requirements and difficulties in antimicrobial testing, identifying and treating these microorganisms poses significant challenges.

**Methods:**

This retrospective study was conducted at Dr. Sami Ulus Maternity and Children’s Health and Diseases Research and Education Hospital, a tertiary care centre in Ankara, Turkey. Blood cultures were screened for *Granulicatella* spp. between January 2005 and January 2017. Clinical and laboratory features of the patients were documented.

**Results:**

During the 12-year study period, 4125 patients with positive blood culture results were investigated. No cases of *G. para-adiacens* or *G. elegans* infection were identified. *G. adiacens* infection was diagnosed in seven patients (five males and two females) representing 0.1%. The mean age of the patients was 79.5 ± 49.8 months (median: 96 months, range: 10–140 months). Six patients had underlying conditions, including congenital heart diseases (two patients), gastrointestinal diseases (two patients), haematological malignancy (one patient), and neurological disorders (one patient). Three patients had bacteraemia, two had central line-related bloodstream infection (CRBSI), one had bacteraemia and pneumonia, and one had infective endocarditis. Four infections were community-acquired and three were healthcare-associated. All patients survived.

**Conclusion:**

Although rare, *G. adiacens* can cause severe infections in children. Clinicians should be particularly vigilant for this pathogen, especially in children with cardiac disease, malignancy, or mucosal disruption, particularly when slow-growing gram-positive cocci are isolated from blood cultures or other sterile sites.

**Supplementary Information:**

The online version contains supplementary material available at 10.1007/s10096-025-05154-0.

## Introduction

Nutritionally variant streptococci (NVS), reclassified as *Abiotrophia defectiva* or *Granulicatella* spp., comprise four species: *A. defectiva*,* G. adiacens*,* G. elegans*, and *G. para-adiacens.* These bacteria are part of the normal microbiota in the upper respiratory, urogenital, and gastrointestinal tracts of humans. They are fastidious gram-positive bacteria that require pyridoxine or thiol supplementation for growth. Because of these requirements, *Abiotrophia* and *Granulicatella* grow in culture as satellite colonies around other bacterial species, such as *Staphylococcus aureus* [1]. The organisms grow poorly on solid media, making them easy to overlook. It is suggested that *Granulicatella* species should be considered in patients where slow-growing alpha-haemolytic streptococci are isolated from blood cultures or other sterile sites [2].

The organisms occasionally penetrate local barriers, causing life-threatening diseases, particularly in patients with predisposing factors. *Granulicatella* spp. most commonly lead to endocarditis and bacteraemia but have also been linked to other infections such as parapharyngeal abscess, tubo-ovarian abscess, mandibular osteomyelitis, and early onset sepsis in paediatric patients [3–6]. In adults, *G. adiacens* has been reported to cause infections in prosthetic joints, vertebral osteomyelitis, and psoas abscess [7–9]. Data on *G. adiacens* infection usually consist of case reports and case series and mostly include adult patients. This report describes the clinical presentations, laboratory characteristics, treatment modalities, and outcomes of *G. adiacens* infections in paediatric patients where data is limited.

## Materials and methods

This retrospective study was conducted at Dr. Sami Ulus Maternity and Children’s Health and Diseases Training and Research Hospital, a tertiary care centre in Ankara, Turkey. Blood cultures were screened for *Granulicatella* spp. between January 2005 and January 2017. Patients aged 1 month to 18 years were included in this study. Information on age, sex, underlying diseases, infectious foci, echocardiographic examinations (if performed), treatment course, and patient outcomes was collected from medical records. Bacteraemia was diagnosed based on the isolation of bacterium in one or more peripheral blood cultures. Hospital-acquired bacteraemia was defined as bacteraemia occurring 48 h or more after hospital admission. Community-acquired infection was defined as bacteraemia occurring within the first 48 h after hospital admission. For suspected central line-related bloodstream infection (CRBSI), paired blood samples were obtained. CRBSI was diagnosed when pathogen growth was detected at least two hours earlier in a blood culture drawn from a central line than that drawn from a peripheral site [10].

Bacterial growth was detected in blood cultures using the BacT/Alert^®^3D automated system (bioMerieux, France). Gram staining of the cultures revealed Gram-positive cocci in chains. Cultures on chocolate agar plates incubated aerobically yielded alpha-haemolytic colonies. Identification of the microorganism and susceptibility testing were performed using the VITEK 2 automated system (bioMerieux, France). Statistical analysis was performed using SPSS v.15.0 for Windows (SPSS, Inc., Chicago, IL, USA).

Ethics committee approval was obtained from Dr. Sami Ulus Maternity and Children’s Education and Research Hospital Ethics Committee (11.09.2024, No: 2024/03) in accordance with the International Guideline for Ethical Review of Epidemiological Studies and Declaration of Helsinki. The informed consent was waived because this study was a retrospective study with review of related data through the electronic medical records.

## Results

During the 12-year study period, 4125 patients with positive blood culture results were reviewed. Seven patients (five males and two females) were diagnosed with *G. adiacens* infection (0.1%), no cases of *G. para-adiacens* or *G. elegans* infection were identified. The mean age of the patients was 79.5 ± 49.8 months (median: 96 months, range: 10–140 months). Six patients had underlying diseases, including congenital heart disease (two patients), gastrointestinal disease (two patients), haematological malignancy (one patient), and neurological disorders (one patient). The frequency of culture positivity was two (Case 1 and 2) in two patients, and three (Case 5) in one patient. Four infections were community-acquired and three were healthcare-associated. The demographic and clinical characteristics of the patients *with G. adiacens* infection are summarised in Table 1.

**Table 1:** Demographic and clinical characteristics of the patients with *G. adiacens* infection

Case	Age/sex	Primary disease	Acquired source	CVL	Clinical situation	Frequency of culture positivity
1	8 years/M	Intestinal lympangiectasia	Health-care	+	CRBSI	2
2	33 months/F	ALL	Health-care	+	CRBSI	2
3	11 years/M	Ulcerative colitis	Community	-	Bacteraemia	1
4	10 years/M	Cerebral palsy	Health-care	+	Bacteraemia	1
5	8 years/M	ASD, VSD, nephrotic syndrome	Community	-	Infective endocarditis	3
6	4 years/F	Dental abscess	Community	-	Bacteraemia	1
7	10 months/M	Tetralogy of fallot	Community	-	Bacteraemia, pneumonia	1

*month, M: male, F: female, ASD: atrial septal defect, VSD: ventricular septal defect,

CVL: central venous line, CRBSI: central line-related blood-stream infection

Echocardiography was performed in three patients (Cases 1, 2, and 5), one patient was diagnosed with infective endocarditis. Three patients had bacteraemia, two had CRBSI, and one had bacteraemia and pneumonia. Five patients received antibiotic treatment without adjunctive treatment. Catheter removal was performed in one of the two patients with CRBSI (Case 1). In the other patient with CRBSI (Case 2), catheter removal was not feasible, but antibiotic lock therapy along with systemic antibiotic treatment eradicated the microorganism. In addition to antimicrobial therapy, aortic valve replacement surgery was performed on the patient with infective endocarditis. Antimicrobial susceptibility tests were performed in Cases 2 and 5. Case 2 was intermediately resistant, whereas Case 5 was resistant to penicillin. Both isolates were sensitive to ceftriaxone. All patients fully recovered. The treatment regimens, durations and outcomes of the patients are summarised in Table 2.

**Table 2:** The treatment regimens, treatment durations and outcomes of the patients with *G. adiacens* infection

Case	Empiric antibiotic treatment	Antibiotic treatment during G. adiacens infection	Treatment duration (day)	Adjunctive treatment	Outcome
1	Ceftriaxone	Ceftriaxone, vancomycin	14, 14	Catheter removal	Cured
2	Ceftriaxone	Ceftriaxone, teicoplanin	14, 7	Antibiotic lock therapy	Cured
3	Ceftriaxone	Ceftriaxone	7	-	Cured
4	Meropenem	Meropenem	38	-	Cured
5	Ceftriaxone, amikacin	Ceftriaxone, amikacin, vancomycin	40, 21, 40	Cardiac surgery	Cured
6	Ampicillin- sulbactam	Ampicillin-sulbactam	10	-	Cured
7	Ampicillin- sulbactam	Ampicillin-sulbactam	7	-	Cured

## Discussion

*Granulicatella* species are uncommon causes of human infections. Despite their rarity, these organisms can cause invasive infections. Most reported isolates were recovered from blood cultures [2]. In one study, which included individuals aged between 15 and 85 years and 302 isolates from blood cultures over six years, *G. adiacens* and *A. defectiva* accounted for 3% of bacteraemia cases due to alfa-haemolytic streptococcus-related species other than *S. pneumoniae*. Seven isolates were identified as *G. adiacens* and two as *A. defectiva* [11]. Pediatric *Granulicatella* bacteraemia is rare compared to adults. Among 97 patients with *G. adiacens*, *G. elegans*, or *A. defectiva* infections, five patients were less than 15 years in a study [12]. The isolation rate of *G. adiacens* was 0.1% in our study. Data on the prevalence of *G. adiacens* bacteraemia in the paediatric population are lacking.

Historically, *Granulicatella* spp. may have been under-reported as culture-negative endocarditis, especially in children. The advent of advanced techniques has made these organisms detectable [13]. As of 2023, the Duke International Society of Cardiovascular Infectious Diseases included *Granulicatella* species as typical infective endocarditis pathogens [14]. *Abiotrophia defectiva* and *Granulicatella* spp. account for approximately 5% of endocarditis cases in adults, with a lower estimated frequency in children [15]. To date, few paediatric cases of infective endocarditis caused by *G*. *adiacens* have been reported [16]. Typically, these bacteria cause endocarditis in children with congenital heart disease [17]. In this study, echocardiography was performed on three children with more than one positive blood culture, and infective endocarditis was diagnosed in a patient with atrial septal defect (ASD) and ventricular septal defect (VSD). Infective endocarditis caused by *Granulicatella* spp. is often more severe than that caused by viridans streptococci. Cardiac vegetations are larger typically with *Granulicatella*, leading to higher frequency of systemic emboli [18, 19]. Valve replacement rates are also higher (around 50%) than those of viridans streptococcal endocarditis, which may be related to delays in diagnosis and treatment [19]. However, a recent study reported similar clinical features, surgery rates, and prognoses for *Granulicatella* and viridans streptococcal endocarditis. These findings were attributed to improvements in microorganism isolation and identification, timely and appropriate antibiotic treatment, and better access to surgical interventions [20].

It is recommended that all patients with *Granulicatella* endocarditis should receive long-term combination therapy. Combination therapy with a cell wall-active agent (e.g. penicillin or vancomycin), and aminoglycosides result in synergistic bactericidal activity [15]. Antibiotic recommendations include penicillin G, ceftriaxone, or vancomycin for six weeks, combined with an aminoglycoside for at least the first two weeks in cases of prosthetic valve endocarditis due to *Granulicatella* spp [19]. Relapse may occur even in cases of endocarditis caused by highly susceptible strains [17]. Our patient with infective endocarditis was treated with combined antimicrobial treatment for six weeks, aortic valve replacement was performed, and no relapse was observed.

*Granulicatella* isolates were primarily found in immunocompromised patients, possibly due to severe neutropenia and an impaired innate immune system at the mucosal barrier, leading to bacterial translocation. A previous study reported that seven of eight cases of bacteraemia caused by *Granulicatella* species (mostly *G. adiacens*) occurred in patients with neutropenia [21]. In our study, one patient had acute lymphoblastic leukaemia and experienced chemotherapy-induced neutropenia and mucositis, which is consistent with the literature. Another predisposing factor identified in our study was dental abscesses. *G. adiacens* constitutes more than 80% of the normal oral cavity flora, often found in dental plaque and endodontic infections [22–24]. Patients without known risk factors can develop infective endocarditis due to invasive procedures such as dental procedures [25]. This may be attributed to the haematogenous spread of the organism from the oral cavity. Similarly, bacteraemia may occur due to the disruption of the gut mucosal barrier in patients with ulcerative colitis [26, 27]. Additionally, hypogammaglobulinemia and impaired immunity likely render patients with intestinal lymphangiectasia susceptible to this infection [28].

The most common causes of CRBSIs are Coagulase-negative staphylococci, *Staphylococcus aureus*, and enterococci [29]. Only one report in the literature documented catheter infection due to *G. adiacens* in a 46 year-old female with acute lymphoblastic leukaemia (ALL), without information on whether the catheter was removed [9]. It remains unclear whether catheter removal is essential in CRBSI caused by *Granulicatella* spp. Given the potential for significant morbidity and mortality, catheter removal was performed in our patient with intestinal lymphangiectasia. However, this was not feasible in another patient with ALL because of the limited access sites. In this case, antibiotic lock therapy was administered. Antibiotic lock therapy can be used to treat CRBSI when catheter salvage is the goal in uncomplicated clinical situations. However, for CRBSIs caused by organisms such as *S. aureus*, *P. aeruginosa*, *Bacillus* spp., *Micrococcus* spp., *Propionibacterium* spp., *Mycobacterium* spp., or fungi, catheter removal is recommended instead of antibiotic lock therapy with catheter retention [30]. We achieved microbiological cure in both patients with systemic antimicrobial treatment.

Isolation of *G. adiacens* can be challenging, making antimicrobial susceptibility testing difficult in clinical laboratories. The recommended method for determining antimicrobial susceptibilities of these organisms is broth microdilution MIC testing using supplemented Mueller-Hinton broth [31]. Novel technologies such as MALDI-TOF mass spectrometry show promise in preventing misidentification and achieving species-level identification of NVS, outperforming the commonly used VITEK 2 system [32]. Approximately 33–67% of *Granulicatella* strains exhibit intermediate resistance (MIC, 0.25–2.0 µg/mL) to penicillin, with some isolates showing high resistance (MIC ≥ 4 µg/mL) [1]. MALDI-TOF mass spectrometry was not available in our hospital. Antimicrobial susceptibility testing could be performed on two isolates in our study. One was defined as intermediately resistant to penicillin, whereas the other was resistant. Both isolates were sensitive to ceftriaxone. A study from the USA tested 132 NVS blood isolates collected between 2008 and 2014. Among the 90 isolates identified as *G. adiacens*, 38.9% were susceptible to penicillin and 43.3% were susceptible to ceftriaxone. Overall, 48.6% of the *G. adiacens* isolates susceptible to penicillin were also susceptible to ceftriaxone. All isolates were susceptible to vancomycin, and none displayed high-level resistance to aminoglycosides [32]. In our study, both isolates were found to be sensitive to teicoplanin. Vancomycin was tested in one patient and was defined as susceptible. While aminoglycosides were not tested, they were administered to the patient with infective endocarditis as part of a combined antimicrobial regimen.

This study had some limitations, primarily due to its retrospective and single-centre nature. The low isolation rate of *G. adiacens* compared with the adult literature may be attributed to the fact that most children had only one set of blood cultures. Additionally, the lack of antimicrobial susceptibility testing for most isolates is a notable limitation. Although no treatment failure was observed, de-escalation was not performed in these cases.

Because *G. adiacens* can cause invasive infections, clinicians must be aware and vigilant. Prompt diagnosis and treatment in children depends on the suspicion and identification of this microorganism by microbiologists. Treatment should be guided by antimicrobial sensitivity results to ensure effective management.

## Electronic supplementary material

Below is the link to the electronic supplementary material.

Supplementary Material 1

## Data Availability

No datasets were generated or analysed during the current study.
