# Supplementary material for: Granulicatella adiacens infections in children: a single-center retrospective study
Source: Eur J Clin Microbiol Infect Dis. 2025 May 8;44(8):1873–8. doi: 10.1007/s10096-025-05154-0 (PMC12321922; doi:10.1007/s10096-025-05154-0)
Supplement: Supplementary file 1 — Supplementary Material 1 [file 10096_2025_5154_MOESM1_ESM.docx]

***G. adiacens* Infections in Children: A Single-center Retrospective Study**

***G. adiacens* Infections in Children**

Turkan Aydın Teke, MD^1^, Ayse Kaman, MD^1^, Fatma Nur Oz, MD^1^, Zeynep Gökce Gayretli Aydın, MD^2^, Hulya Seker Yıkmaz, MD^3^, Gulsum Iclal Bayhan, MD^4^

^1^ Department of Pediatric Infectious Disease, Ankara Etlik City Hospital, Ankara, Türkiye

^2^ Department of Pediatric Infectious Disease, Faculty of Medicine, Karadeniz Technical University, Trabzon, Türkiye

^3^ Department of Pediatrics, Faculty of Medicine, Ankara Yıldırım Beyazıt University, Ankara City Hospital, Ankara, Türkiye

^4^ Department of Pediatric Infectious Disease, Faculty of Medicine, Ankara Yıldırım Beyazıt University, Ankara City Hospital, Ankara, Türkiye.

**Address for correspondence:** Turkan Aydın Teke, MD, Department of Pediatric Infectious Disease, Ankara Etlik City Hospital, 06170, Ankara, Turkiye

**Telephone number:** 05058580156

**E-mail:** turkanteke@gmail.com

The authors have no funding or conflicts of interest to disclose.

**Key words:** *Granulicatella adiacens*, bacteraemia, endocarditis, central line-related bloodstream infection, children.
